# Health Outcomes Following the Health‐Promoting “Reflective STRENGTH‐Giving Dialogue” Intervention Among Community‐Dwelling Older Adults

**DOI:** 10.1111/scs.70280

**Published:** 2026-06-10

**Authors:** Cecilia Åberg, Catharina Gillsjö, Mia Berglund, Jenny Hallgren

**Affiliations:** ^1^ School of Health Sciences University of Skövde Skövde Sweden; ^2^ University of Rhode Island College of Nursing Kingston Rhode Island USA

**Keywords:** dialogue, healthy aging, intervention, older adults, quasi‐experimental design, well‐being

## Abstract

**Background:**

Effective health‐promoting interventions can support older adults in maintaining and regaining good health and can foster healthy aging. The Reflective STRENGTH‐Giving Dialogues (STRENGTH) intervention was created to optimise a holistic healthcare delivery that supports older adults in learning to live with long‐term musculoskeletal pain. Findings from previous studies indicate that the dialogues contributed to an increased sense of well‐being and had an immediate pain‐alleviating effect. The aim of this extended study was to explore the health effects of the STRENGTH intervention among older community‐dwelling adults living with long‐term health problems.

**Methods:**

Older adults (*n* = 47) in Sweden completed questionnaires inquiring about levels of well‐being, impact of health problems on daily life, depression symptom occurrences, health‐related quality of life, physical performance, and consumption of care using a quasi‐experimental design. Descriptive statistics were used to explain and compare results within groups. Data were collected before, during, and after the STRENGTH intervention in autumn 2017 and spring 2018. It consisted of health‐care professionals engaging in recurrent dialogues to guide and support older adults in ways that increase sense of well‐being, joy, strength, and meaning in life through carrying out small and large life projects.

**Results:**

According to self‐reports, the STRENGTH intervention had immediate positive effects on perceived well‐being and health problems. From a longitudinal perspective, although no significant differences in health outcomes were found based on comparisons of baseline and follow‐up data within groups, positive effects were shown.

**Conclusions:**

To contribute to healthy aging, HCPs need favourable conditions for conversations in care encounters, extensive knowledge about the importance and potential of dialogues, and an understanding of how to integrate dialogues into health and social care.

## 1 Introduction

The World Health Organization (WHO) is centring its work on issues related to aging. And from 2015 to 2030 a special focus is on the promotion of healthy aging, which can be enhanced through opportunities that enable people to be and do what they value in their lives [1]. Effective health‐promoting interventions can support older adults in maintaining and regaining good health and can foster healthy aging [2]. This is consistent with the description of health according to Dahlberg et al. [3], which entails a person's sense of well‐being and their ability to carry out the minor and major projects they valued throughout their lives.

Health‐promoting interventions for community‐dwelling older adults often focus on fall prevention [4, 5, 6]. Other interventions target specific themes determined in advance, such as eating habits and physical activity [7], or concentrate on a certain type of health problem, like depressive symptoms [8]. A significant number of management programmes for different single chronic conditions have been developed, but this has led to a deficiency in the development of care for older adults living in a complex situation with several contemporary, long‐term health problems [9]. Needs, such as engaging in meaningful everyday activities and social interaction, are frequently unaddressed in older adults [10]. Indeed, despite the growing number of older adults living with long‐term health problems, the provision of health care is not as comprehensive as needed [11, 12]. Given these circumstances, the content and health effects of health care services need to be strengthened to optimally meet this population's needs for healthier aging experiences [13].

The Reflective STRENGTH‐Giving Dialogues (STRENGTH) intervention was created to optimise a holistic healthcare delivery that supports older adults in learning to live with long‐term musculoskeletal pain [14]. During recurrent dialogues, a tactful and challenging approach is used by the health‐care professional (HCP) to increase the older adult's awareness of the possibilities and choices available for enhancing their life despite their long‐term pain. Findings from previous studies on the STRENGTH intervention indicate that the dialogues contributed to an increased sense of security, strength, courage, confidence [15], and well‐being and had an immediate pain‐alleviating effect [16]. However, no significant differences were found on the occurrence or level of depression of the older adults living with long‐term musculoskeletal pain, according to the Geriatric Depression Scale (GDS)‐20 instrument. Further research to explore the effects of the STRENGTH method into other contexts and using additional quantitative data collection methods is needed. The current STRENGTH intervention, with the addition of the reflective SelfSTRENGTH app, focuses on community‐dwelling older adults living with long‐term health problems. In addition, instruments measuring HRQoL, physical performance, and consumption of care have been added to further explore the effects of the intervention.

The aim of this study was to explore the health effects of the STRENGTH intervention among older community‐dwelling adults living with long‐term health problems.

The following research questions were posed:

1. What, if any, immediate effect does each reflective STRENGTH‐giving dialogue within a STRENGTH intervention have on the self‐reported level of impact that health problems have on the daily life and well‐being of older community‐dwelling adults?
2. What, if any, longitudinal effects do continuous STRENGTH‐giving dialogues within a STRENGTH intervention have on the self‐reported level of impact that health problems have on the daily life and well‐being of older community‐dwelling adults?
3. What, if any, longitudinal effects do continuous STRENGTH‐giving dialogues within a STRENGTH intervention have on the health of older community‐dwelling adults?

## 2 Materials and Methods

### 2.1 Study Design

A quasi‐experimental pretest–posttest no‐treatment control group design was applied. The older adults in the control group received care as usual, while the older adults in the intervention group received the intervention involving approximately 10 (6–10) Reflective STRENGTH‐giving dialogues during a 14‐week period.

### 2.2 Participants and Procedure

**FIGURE 1:**
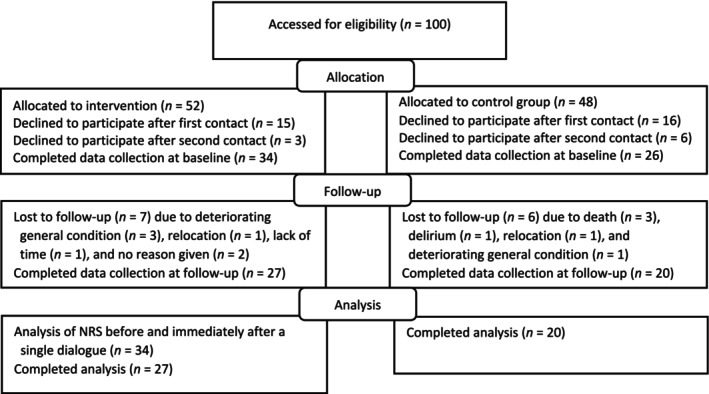
Flow chart of study participants.

Potential participants (*n* = 100) were identified by HCPs in municipal care according to a strategic sample procedure, avoiding participants who had a professional relationship with the HCP providing the intervention. Participants had to meet the following eligibility criteria: community‐dwelling, age 65 or older, Swedish‐speaking, with persistent or regularly recurring health problems classified as long term (experienced for at least 6 months prior to the study). Names and contact details were reported to the development manager, who arranged the division for control and intervention group members, considering their geographic areas to prevent a spillover effect. The older adults, who resided at home and in assisted living facilities in the South of Sweden, were informed about the study through a phone call from the development manager or in person by the HCP. Next, the first author scheduled an appointment with the participants, and both oral and written consent was obtained. Of the 52 older adults in the intervention group who met the inclusion criteria, 34 agreed to participate in the study. For the control group, 26 of the 48 who met the inclusion criteria agreed to participate (see Figure 1). Reasons for not participating were poor strength and lack of interest in or need for dialogues.

### 2.3 Data Collection Procedure

Data were collected via face‐to‐face contact by the first author on two occasions: baseline data were gathered 1–2 weeks before the 14‐week intervention, and follow‐up data were gathered 1–2 weeks after the intervention. The intervention was conducted during two periods (Period I—September to December 2017; Period II—February to May 2018), so the intervention group's baseline data were collected in August 2017 and January 2018, and follow‐up data were collected in December 2017 and May 2018. The control group's baseline data were collected with the same time span in September 2017 and February 2018 and follow‐up data were collected in January and June 2018. Data collection, which took place almost exclusively in the participants' homes, began with gathering demographic data, followed by interviews (intervention group only) and surveys administered in the following order: Numeric Rating Scale (NRS), Geriatric Depression Scale (GDS‐20), RAND‐36 Item Health Survey, and Short Physical Performance Battery (SPPB‐S). Characteristics of the study participants are provided in Table 1. NRS data were collected in the intervention group by the HCP before and after each dialogue.

**TABLE 1:** Baseline characteristics.

Baseline characteristics	Intervention group		Control group	
	Baseline (*n* = 34)	Follow‐up (*n* = 27)	Baseline (*n* = 26)	Follow‐up (*n* = 20)
*Age*				
Year, mean (SD)	86.3 (6.53)	87.0 (6.10)	82.2 (7.88)	83.4 (6.90)
Range (min–max)	74–96	75–96	66–94	67–93
65–74, *n* (%)	2 (5.8)	0 (0.0)	4 (15.3)	2 (10.0)
75–84, *n* (%)	9 (26.4)	8 (29.6)	8 (30.7)	6 (30.0)
85+, *n* (%)	23 (67.6)	19 (70.3)	14 (53.8)	12 (60.0)
Female gender, *n* (%)	23 (67.6)	20 (74.0)	19 (73.0)	13 (65.0)
Male gender, *n* (%)	11 (32.3)	7 (25.9)	7 (26.9)	7 (35.0)
*Living situation*				
Single, *n* (%)	32 (94.1)	26 (96.2)	21 (80.7)	17 (85.0)
*Housing situation*				
Ordinary, *n* (%)	24 (70.5)	18 (66.6)	19 (73.0)	15 (75.0)
Assisted living facility, *n* (%)	10 (29.4)	9 (33.3)	7 (26.9)	5 (25.0)
*Medication*				
Anxiety/sleeping, *n* (%)	16 (53.3)		9 (37.5)	
Pain, *n* (%)	26 (78.8)		15 (60.0)	
Antidepressant *n* (%)	10 (34.5)		4 (16.7)	
*Help situation*				
Interventions according only to the Social Services Act (SoL), *n* (%)	3 (8.8)	2 (7.4)	5 (19.2)	5 (25.0)
Interventions according only to the Health and Medical Service Act (HSL), *n* (%)	0 (0.0)	0 (0.0)	2 (7.6)	1 (5.0)
Interventions according to both SoL and HSL, *n* (%)	31 (91.1)	25 (92.6)	19 (73.0)	14 (70.0)
Weekly visits from home care providers, mean (SD)	22.35 (11.75)		17.23 (10.05)	
Alarms to home care providers during 2 weeks, mean (SD)	4.30 (8.53)	2.17 (4.72)	1.55 (3.99)	1.35 (3.58)
*Health problems*				
Number of self‐reported health‐problems, mean (SD)	5.24 (2.40)	5.24 (2.40)	6.04 (2.63)	6.04 (2.63)
Occurrence of health problems body function‐related (ICF) *n* (%)				
Mental functions	22 (64.7)	22 (64.7)	18 (69.2)	18 (69.2)
Sensory functions	22 (64.7)	22 (64.7)	20 (76.9)	20 (76.9)
Pain	28 (82.3)	28 (82.3)	18 (69.2)	18 (69.2)
Functions of cardiovascular, haematological, immunological, and respiratory systems	19 (55.8)	19 (55.8)	15 (57.6)	15 (57.6)
Functions of digestive, metabolic, and endocrine systems	10 (29.4)	10 (29.4)	11 (42.3)	11 (42.3)
Genitourinary and reproductive functions	6 (17.6)	6 (17.6)	4 (15.3)	4 (15.3)
Neuromusculoskeletal and movement‐related functions	28 (82.3)	28 (82.3)	22 (84.6)	22 (84.6)
Functions of skin and related structures	7 (20.5)	7 (20.5)	10 (38.4)	10 (38.4)

### 2.4 Measures

The majority of the questionnaires were used in the previous STRENGTH intervention [16], which is why they were chosen in this study as well. As there were observations in the previous study regarding increased physical activity after the intervention, a measuring instrument was added.

#### 2.4.1 Evaluating Well‐Being and the Impact of Long‐Term Health Problems on Daily Life Using the Numeric Rating Scale (NRS)

The NRS is a verbal instrument used in most settings for assessment of pain intensity [17]. In this study, the 0–10 NRS was used to evaluate well‐being (0 = lowest level, 10 = highest level) and impact of long‐term health problems on daily life (0 = no impact, 10 = highest impact) immediately before and after each STRENGTH dialogue, as well as before and after the intervention. Participants were asked to rate how they felt in the moment, reflecting their current well‐being and impact of health problems.

#### 2.4.2 Questionnaires

The GDS was originally developed as a 30‐item instrument to assess the depression levels of older adults [18]. The 15‐item GDS short form has high sensitivity and specificity for diagnosing depression in the oldest subjects [19]. Five items related to sleeping habits, anxiety, pain, and worries about illness in daily living were added to the modified GDS‐20 Swedish version [20]. This 20‐item scale has dichotomous answer alternatives (yes or no), with a score of 0–5 indicating that depression is unlikely and a score of 6 or more indicating that depression is suspected.

The RAND‐36 Item Health Survey, developed during the 1980s and intended as a generic measure of HRQoL in adults, measures eight health domains (Table 3) preferably as they were experienced during the 4 weeks prior to administration of the survey [21]. The answers to the RAND‐36 questions are compiled into eight dimensional points according to a standardised calculation procedure, through which every item is linearly transformed to a 0–100 possible range. Higher scores represent higher rated levels of health status [21]. The RAND‐36 has been translated into more than 50 languages and has been validated extensively [21, 22]. This assessment was translated and culturally adapted into Swedish [23].

Finally, the Short Physical Performance Battery (SPPB) is an objective instrument for measuring lower extremity function that was developed to identify the emergence of a disability in older adults [24]. The SPPB is reliable and valid for evaluating community‐dwelling older adults [25]. The maximal score is 12 points, with higher scores indicating a higher level of physical functioning. The Swedish version (SPPB‐S) was administered in this study according to the standard procedures previously published by Guralnik et al. [24].

#### 2.4.3 Consumption of Care

Self‐perceived health problems were reported by the older adults to the first author at the baseline point. Help situations according to the Social Services Act [SoL] [26] and the Health and Medical Services Act [HSL] [27] were reported by the older adults both at the baseline and follow‐up points.

### 2.5 Data Analysis

Descriptive statistics were used to present baseline data. The Shapiro–Wilk statistic was used to test normality. As the distributions were not normal, the Wilcoxon signed‐rank test was used to explore differences in the baseline and follow‐up data and changes over time within individuals. Cohen's d was used to present effect sizes. A *p* < 0.05 was considered statistically significant. Cronbach's alpha indicated critically low internal consistency for the GDS‐20 (*α* = 0.42), suggesting low reliability in the present sample, while the RAND‐36 demonstrated good internal consistency (*α* = 0.81). The SPSS Statistical Package for Social Sciences version 27.0 for Windows was used for statistics and data handling.

### 2.6 Intervention

**FIGURE 2:**
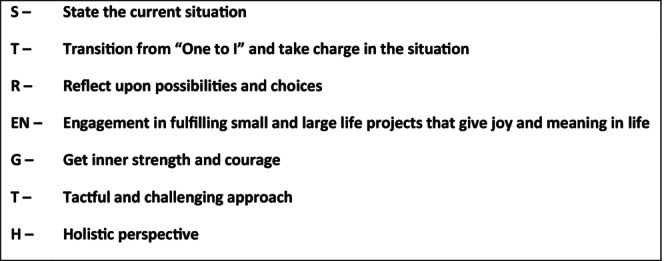
STRENGTH key dimensions.

The 10‐session STRENGTH intervention consisted of 60‐min dialogues, about once a week, with a specific and educated HCP (occupational therapist, physiotherapist, or registered nurse) in the home environment. The dialogues, grounded in a lifeworld perspective, aim to guide and support the older adults in ways that increase a person's sense of well‐being, joy, strength, and meaning in life by carrying out small and large life projects [14]. Following a tactful and challenging approach, reflection‐supporting questions and tools were employed throughout the intervention, and focus was on exploring the older adults' own goals rather than goals determined by the HCP. The key dimensions in STRENGTH are illustrated in Figure 2. In the SelfSTRENGTH app, used by the older person or with support by the HCP, reflection takes place on one's mood, goals, and orientation in life. It also aims to stimulate activity that enables meaningful life projects to be achieved [28].

#### 2.6.1 Education and Quality Control of Intervention

To enhance the delivery of a consistent intervention, the second and third authors educated the HCP, who had at least one year's experience in home health care, on the STRENGTH intervention method for a total of 3 days and supervised them every fourth week (four times in total). Education focused on physical, psychological, and existential issues in older adults and the purpose and content of STRENGTH, along with utilisation of reflection‐supporting tools, such as pictures, booklets, and the SelfSTRENGTH app.

## 3 Results

Questionnaire baseline data were collected from 60 older adults (34 from intervention group and 26 from control group), and questionnaire follow‐up data were collected from 47 older adults (27 from intervention group and 20 from control group). In follow‐up, seven participants in the intervention group were lost due to deteriorating health conditions (*n* = 3), relocation (*n* = 1), lack of time (*n* = 1), and no reason given (*n* = 2). One participant declined further participation after six dialogues as she did not feel she needed additional dialogues, but she still wanted to be included in the data collection after the intervention. In the control group, six participants were lost due to death (*n* = 3), delirium (*n* = 1), relocation (*n* = 1), and deteriorating health condition (*n* = 1). Most participants in both groups were female (intervention group 23 [67.64%] and control group 18 [69.23%]); moreover, most participants were single (intervention group 32 [94.1%] and control group 21 [80.8%]). Some differences existed between the groups as well. The mean age was 86.38 (±6.53) and 82.27 (±7.88) in the intervention and control groups, respectively. The intervention group accounted for a higher proportion of care consumption in the form of medicine, safety alarms, and weekly visits from home care providers. Furthermore, a higher proportion resided in assisted living facilities in the intervention group. Although the control group indicated higher numbers of self‐reported health problems, the occurrence of health problems related to body functions were more dominant in most domains according to the International Classification of Functioning, Disability, and Health (ICF; [29]) in the intervention group (see Table 1).

### 3.1 Immediate Effects on Impact of Health Problems on Daily Life and Well‐Being After Each Single STRENGTH Dialogue Using the NRS

**FIGURE 3:**
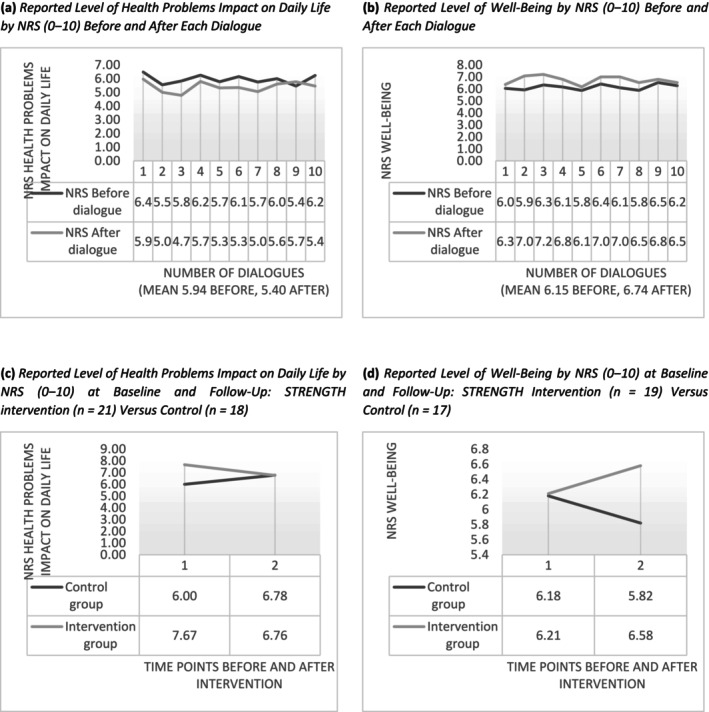
Reported levels of NRS using Wilcoxon signed‐rank test.

The total level of impact that health problems had on daily life was reported to be lower (5.40 [0.38]) immediately after each STRENGTH dialogue compared to the level reported before each dialogue (5.94 [0.32]) on a 0–10 NRS. For reported level of health problems impact on daily life by NRS (0–10) before and after each dialogue, see Figure 3a. The total level of well‐being reported was higher after each STRENGTH dialogue (6.74 [0.33]) compared to before each dialogue (6.15 [0.23]). For reported level of well‐being by NRS (0–10) before and after each dialogue, see Figure 3b.

Significant differences according to Wilcoxon signed‐rank test in reported level of impact of health problems on daily life by NRS (0–10) before and after each dialogue were found for dialogue 2 (*p* = 0.013) (*d* = 0.54), dialogue 3 (*p* = 0.018) (*d* = 0.53), and dialogue 10 (*p* = 0.047) (*d* = 0.59). Significant differences in reported level of wellbeing by NRS (0–10) before and after each dialogue were found in dialogue 2 (*p* = < 0.001) (*d* = 0.98), dialogue 3 (*p* = 0.002) (*d* = 0.68), dialogue 4 (*p* = 0.044) (*d* = 0.43), dialogue 6 (*p* = 0.046) (*d* = 0.40), and dialogue 7 (*p* = 0.004) (*d* = 0.72); see Table 2.

**TABLE 2:** Baseline and follow‐up scores within each single dialogue, NRS.

Variables	Intervention group		*p* *
	Baseline	Follow‐up	
	Mean (SD)	Mean (SD)	
*NRS well‐being within each single dialogue*			
Dialogue 1 (*n* = 27)	6.04 (1.97)	6.37 (1.47)	0.208
Dialogue 2 (*n* = 25)	5.92 (1.80)	7.08 (1.77)	**< 0.001**
Dialogue 3 (*n* = 24)	6.33 (1.76)	7.21 (1.35)	**0.002**
Dialogue 4 (*n* = 25)	6.16 (1.70)	6.80 (1.38)	**0.044**
Dialogue 5 (*n* = 23)	5.87 (2.05)	6.17 (2.05)	0.196
Dialogue 6 (*n* = 22)	6.41 (1.70)	7.00 (1.15)	**0.046**
Dialogue 7 (*n* = 21)	6.10 (1.44)	7.00 (1.37)	**0.004**
Dialogue 8 (*n* = 17)	5.88 (2.02)	6.53 (2.06)	0.077
Dialogue 9 (*n* = 15)	6.53 (1.55)	6.80 (1.65)	0.314
Dialogue 10 (*n* = 15)	6.27 (2.34)	6.53 (2.35)	0.297
*NRS Impact of health problems within each single dialogue*			
Dialogue 1 (*n* = 25)	6.48 (0.53)	5.96 (2.31)	0.171
Dialogue 2 (*n* = 22)	5.55 (1.89)	5.00 (2.13)	**0.013**
Dialogue 3 (*n* = 23)	5.83 (2.03)	4.78 (2.17)	**0.018**
Dialogue 4 (*n* = 24)	6.25 (1.80)	5.79 (1.81)	0.185
Dialogue 5 (*n* = 22)	5.77 (2.28)	5.32 (1.91)	0.127
Dialogue 6 (*n* = 20)	6.15 (2.13)	5.35 (2.00)	0.069
Dialogue 7 (*n* = 20)	5.75 (2.04)	5.05 (2.37)	0.154
Dialogue 8 (*n* = 15)	6.00 (2.00)	5.60 (1.95)	0.141
Dialogue 9 (*n* = 13)	5.46 (2.10)	5.77 (2.20)	0.234
Dialogue 10 (*n* = 13)	6.23 (2.12)	5.46 (2.40)	**0.047**

*Notes:* The bold values indicate statistically significant differences (*p* < 0.05).

* Wilcoxon signed‐rank test.

### 3.2 Longitudinal Effects on Impact of Health Problems on Daily Life and Well‐Being Before and After STRENGTH Intervention Using NRS

The level of impact of health problems on daily life was reported to be lower after the STRENGTH intervention (6.76 [2.16] *p* = 0.104) compared to before it started (7.67 [1.77]) on a 0–10 NRS (see Figure 3c). The level of well‐being reported was higher after the intervention (6.58 [1.61] *p* = 0.401) compared to before it started (6.21 [2.41]; see Figure 3d). Opposite conditions prevailed in the control group: the level of impact of health problems on daily life reported was higher at the point of follow‐up (6.78 [2.39] *p* = 0.316) than at the baseline (6.00 [2.87]). Furthermore, the follow‐up level of well‐being in the control group was reported to be lower (5.82 [2.12] *p* = 0.915) than the baseline level (6.18 [2.65]). However, no significant differences were found at follow‐up compared to baseline within groups; see Table 3.

**TABLE 3:** Baseline and follow‐up scores, GDS‐20, SPPB‐S, RAND‐36, NRS.

Variables	Intervention group (*n* = 27)		Within group	Control group (*n* = 20)		Within group
	Baseline	Follow‐up	*p*	Baseline	Follow‐up	*p*
	Mean (SD)	Mean (SD)		Mean (SD)	Mean (SD)	
*GDS‐20, total*	6.67 (3.18)	6.15 (2.67)	0.221	6.05 (4.01)	7.05 (4.01)	0.078
Depression can be suspected, *n* (%)	16 (59.25)	15 (55.55)	0.564	9 (45.00)	12 (60.00)	0.083
*SPPB‐S, total*	3.59 (2.25)	3.78 (2.50)	0.551	3.10 (2.33)	3.15 (2.23)	0.916
Standing balance performance	1.85 (1.48)	2.04 (1.60)	0.468	1.55 (1.35)	1.60 (1.14)	0.794
Walking speed	1.33 (0.87)	1.37 (0.88)	0.796	1.15 (0.74)	1.10 (0.85)	0.655
Sit‐to‐stand test	0.41 (0.50)	0.41 (0.50)	1.000	0.40 (0.59)	0.40 (0.59)	1.000
*RAND‐36*						
Physical functioning	21.11 (19.91)	12.22 (11.20)	**0.006**	27.75 (21.36)	21.35 (20.05)	**0.009**
Role functioning/physical	32.40 (31.63)	40.74 (32.63)	0.200	33.75 (30.64)	30.00 (33.04)	0.748
Pain (bodily pain)	47.12 (24.68)	48.61 (24.45)	0.536	67.00 (27.60)	64.00 (31.53)	0.552
General health	47.03 (24.97)	49.44 (26.50)	0.695	57.00 (20.67)	59.50 (19.72)	0.886
Energy/fatigue (vitality)	48.33 (24.05)	50.00 (19.75)	0.695	61.25 (20.44)	52.00 (21.42)	**0.021**
Social functioning	57.40 (29.26)	64.81 (26.63)	0.195	58.12 (30.15)	55.62 (24.15)	0.800
Role functioning/emotional	76.54 (34.36)	87.65 (30.86)	0.131	86.66 (27.35)	68.33 (41.14)	**0.047**
Emotional well‐being (mental health)	73.77 (19.16)	73.77 (18.93)	0.613	75.60 (22.84)	68.33 (20.48)	**0.046**
Health transition score	33.33 (25.94)	43.51 (24.60)	0.107	48.75 (27.47)	47.50 (26.77)	0.739
*NRS*						
Impact of health problems on daily life^a^	7.67 (1.77)	6.76 (2.16)	0.104	6.00 (2.87)	6.78 (2.39)	0.316
Well‐being^b^	6.21 (2.41)	6.58 (1.61)	0.401	6.18 (2.65)	5.82 (2.12)	0.915

^a^ Intervention *n* = 21/control *n* = 18.

^b^ Intervention *n* = 19/control *n* = 17.

### 3.3 Longitudinal Health Effects of STRENGTH Intervention and Controls

In GDS‐20, improved results in the intervention group and deteriorated results in the control group at follow‐up compared to baseline were observed. SPPB‐S showed improvement in both groups, although not significant. In RAND‐36, physical functioning was reported significantly lower in both intervention (*p* = 0.006) (*d* = 0.61) and control (*p* = 0.009) (*d* = 0.63) groups, while general health was reported higher in both groups at follow‐up compared to baseline. In energy/fatigue [vitality] and role functioning/emotional, the control group reported significantly lower results (*p* = 0.021 and *p* = 0.047) (*d* = 0.58 and *d* = 0.46), while the intervention group reported improved results, although not significant. Regarding emotional well‐being (mental health), the control group reported significantly lower results (*p* = 0.046) (*d* = 0.48), while the intervention group reported no difference at follow‐up. In three of the health domains (role functioning/physical, pain, and social functioning), the levels were improved in the intervention group, but deteriorating results were found in the control group at follow‐up compared to baseline. The health transitions score in RAND‐36 was reported higher in the intervention group and lower in the control group at follow‐up compared to baseline, but no significant improvement was observed over time (*p* = 0.086; see Table 3).

## 4 Discussion

STRENGTH has positive immediate effects in the form of a reduction in the level of impact that health problems have on daily life and increased well‐being after each single dialogue. No significant longitudinal positive health outcomes were found when comparing baseline to follow‐up, despite observed positive trends in the intervention group contrasting with deterioration in the control group. From a longitudinal perspective, no significant differences in NRS at follow‐up compared to baseline within groups were found, although the impact of health problems was reported to be lower, and the level of well‐being was reported higher after the STRENGTH intervention compared to before the intervention. Opposite conditions prevailed in the control group. Similar positive effects from the STRENGTH intervention were shown in older adults living with musculoskeletal pain [16]. The repeated measures of the impact of health problems on daily life and the sense of well‐being may have been influenced by some difficulties that the older adults expressed in scoring during data collection. The NRS was validated for assessment of pain intensity [30], which was not the target in this study. During the qualitative interviews, the older adults expressed [31] that living with several health problems is like living with a body in constant change, where the body's ability and sense of well‐being varies over time and in relation to the intensity of each health problem. To capture these immediate experiences, the NRS was used to assess well‐being and the impact of health problems in the moment, before and after each STRENGTH dialogue. Although participants' health states fluctuate throughout the day, this approach provides a straightforward and interpretable measure of short‐term effects. An alternative approach could have been to ask participants to rate their well‐being and the impact of health problems based on an average over the last 24 h before and after the entire intervention. This might have reduced the influence of moment‐to‐moment fluctuation, but it could also have introduced recall bias or required participants to reflect on experiences that were difficult to remember. Internal validity refers to the extent to which the intervention can be considered responsible for the results and changes [32]. The attention given to the older persons in the intervention group could pose a threat to this validity. Estimating NRS before and after each dialogue in the presence of the HCP who conducted the dialogues may also introduce bias. However, the critical views expressed by the older persons in the intervention group, with expectations that the dialogues would lead to something more than ordinary conversations, suggest an awareness that attention alone was not sufficient to improve health outcomes. Participants in the control group also received attention when asked about their participation in the study and during data collection, which occurred twice. This may have prompted reflections about their health and positively influenced them.

Given the low internal consistency and limited statistical power, the GDS‐20 findings are reported for completeness only and primarily serve to highlight methodological limitations rather than intervention‐related outcomes. In SPPB‐S, there was improvement in both groups. This finding contrasts with the RAND‐36 results for physical functioning, which showed a significant decline in both the intervention (*p* = 0.006) and control (*p* = 0.009) groups at follow‐up compared to baseline. The significant decline in physical functioning observed in both the intervention and control groups suggests that this change is unlikely to be attributable to the intervention itself. Instead, it may reflect the expected decline associated with aging and progressive health problems in this population, along with external factors. A reduction in physical functioning over time may be anticipated, even in the presence of supportive interventions. When collecting the data, the older persons provided a self‐assessment of their health problems, which was challenging because these problems were integrated into their daily lives. However, after completing the written questionnaires, more health problems emerged when they had to respond to direct questions. This suggests that questionnaires can be a valuable complement to self‐assessments. The informants reported to a greater extent at baseline that they were not that limited in physically strenuous activities, such as carrying bags and walking up stairs and around the block but rated it worse in the follow‐up. This pattern may indicate an overestimation of physical functioning at baseline, with functional limitations becoming more apparent at follow‐up when the participants were prompted to reflect on specific physical tasks. Therefore, it is possible that the intervention made the participants more aware of their symptoms, or that merely by asking the participants about their symptoms, the researcher shed light on the phenomenon itself. Despite the decline in physical functioning, improvements over time were observed in several other RAND‐36 health domains in the intervention group (role functioning/physical, pain, energy/fatigue [vitality], social functioning, role functioning/emotional, and health transition score), suggesting that the intervention may have supported aspects of perceived health and well‐being. Results from the qualitative interviews [31, 33] revealed that the older adults expressed that their health problems are difficult to influence, but the STRENGTH dialogues are helpful in formulating realistic goals and are perceived as a starting point in changing their life situations. This shows that, although some variables can be difficult to influence based on the health situation of the older adults, it is possible to influence their attitude towards life.

Even with an intervention designed from a clear theoretical base, there is no guarantee that the intended providers will be prepared, able, or sufficiently skilled to deliver the intervention as designed [34]. Fidelity was strengthened through active participation in the educational programme and regular supervision, during which the HCP received support and feedback to ensure that STRENGTH was carried out as intended. The observed lack of significant longitudinal effects may in part reflect limitations in intervention fidelity. Although the SelfSTRENGTH app and other reflection tools were available, their use may have been constrained by the HCPs' comfort with the use, as well as the older adults' willingness or ability to engage with digital tools. Consequently, participants may not have used the tools to their full extent, which could have influenced the magnitude of observed effects over time.

After all, it is relevant to question whether 10 dialogues are needed. Results from a previous study on preventive home visits to older adults showed that insight into enabling the participants to take part in social contexts was provided during only one visit from a HCP [35]. However, each STRENGTH dialogue has a value in itself with immediate effect and seems to ease the moment. Qualitative studies regarding STRENGTH [15, 33] imply that the dialogues ease the pain for some time. The opportunity to reflect upon life differs from ordinary superficial talks and is viewed as a starting point and a push in finding new meaning in life. The ability to find meaning in life is essential [36, 37]. Therefore, it is important that reflective, in‐depth dialogues that contain what a person is able to and wants to do or be are given a role and take space in health and social care, where both recovery and new strength can be provided.

Limitations of the study include a small follow‐up sample size and a non‐randomised design, which may increase the risk of Type II errors and limit internal validity. It is difficult to match older adults since they are not a homogenous group. Conducting the intervention in another municipality to recruit an increased number of older persons could jeopardise validity due to potential differences in history, organisational factors, and sample characteristics [32]. Descriptive statistics within individuals and in between groups were, therefore, used. Reliability is strengthened by the fact that all data at baseline and follow‐up were collected by one person (CÅ). Further, to increase the validity regarding dropouts, only persons who provided data both at baseline and follow‐up were included in the statistical calculations. Being one's own control with a repeated measure, along with a control group recruited to prevent geographical spillover, is also considered a strength in the study.

### 4.1 Conclusion and Implication for Practice

The study supports that the STRENGTH intervention contributes to increased health and well‐being among older community‐dwelling adults living with long‐term health problems. Immediate positive effects were illustrated although no significant longitudinal positive health outcomes were observed. Living with long‐term health problems often entails the need for support in daily living. STRENGTH dialogues are meaningful for health, well‐being, and inner strength, even if effects are short‐term. To contribute to healthy aging, HCPs need favourable conditions for conversations in care encounters, extensive knowledge about the importance and potential of dialogues, and an understanding of how to integrate dialogues into health and social care. Regarding the SelfSTRENGTH app, several barriers were identified. These challenges highlight that successful implementation of digital tools requires more time and resources spent in the design stage to ensure a user‐friendly and accessible application. Future research should explore strategies to overcome these barriers and enhance digital participation among older adults. Additionally, while this study highlights the potential of digital support through the SelfSTRENGTH app, it is important to note that the majority of participants were aged 85 and older and had very limited prior experience using tablets or apps. This limits the transferability of the findings to younger or more digitally experienced older adults. At the same time, including this typically underrepresented group provides valuable insights into the challenges and requirements for supporting digital participation among the oldest and most vulnerable adults, emphasizing the need for user‐friendly and accessible digital solutions.

## Author Contributions

Study concept and design: Cecilia Åberg, Catharina Gillsjö, Mia Berglund and Jenny Hallgren. Acquisition of data: Cecilia Åberg. Analysis and interpretation of data: Cecilia Åberg and Jenny Hallgren. Drafting on the manuscript: Cecilia Åberg. Critical revision of the manuscript for important intellectual content: Cecilia Åberg, Catharina Gillsjö, Mia Berglund and Jenny Hallgren. All authors meet the criteria for authorship. All authors read and approved the final manuscript.

## Funding

This work was supported by the School of Health Sciences, University of Skövde, Sweden; the Skaraborg Institute for Research and Development; Sparbanksstiftelsen Lidköping; the Gösta Svensson Foundation; and the Agneta Prytz‐Folke and Gösta Folke Foundation [2016‐00023].

## Ethics Statement

All participants were given written and oral information about the study, from the development manager/HCP initially and then from the first author at the time of data collection. Participation in the study was fully voluntary, and all older adults were informed that they were allowed to withdraw at any time during the study, without explanation. The study was approved by the Regional Ethical Review Board in Gothenburg (295‐17). The authors have checked to make sure that our submission conforms as applicable to the Journal's statistical guidelines. The authors affirm that the methods used in the data analyses are suitably applied to their data within their study design and context, and the statistical findings have been implemented and interpreted correctly.

## Conflicts of Interest

The authors declare no conflicts of interest.

## Data Availability

The data that support the findings of this study are available on request from the corresponding author. The data are not publicly available due to privacy or ethical restrictions.
